# Unsupervised neurobiology-driven stratification of clinical heterogeneity in depression

**DOI:** 10.1192/j.eurpsy.2023.1279

**Published:** 2023-07-19

**Authors:** F. Colombo, F. Calesella, B. Bravi, L. Fortaner-Uyà, C. Monopoli, E. Maggioni, E. Tassi, R. Zanardi, F. Attanasio, I. Bollettini, S. Poletti, F. Benedetti, B. Vai

**Affiliations:** 1 University Vita-Salute San Raffaele; 2 Division of Neuroscience, Psychiatry and Clinical Psychobiology Unit, IRCCS San Raffaele Hospital; 3 Department of Electronics Information and Bioengineering, Politecnico di Milano; 4 Department of Neuroscience and Mental Health, Fondazione IRCCS Ca’ Granda Ospedale Maggiore Policlinico; 5 Mood Disorders Unit, IRCCS San Raffaele Hospital, Milan, Italy

## Abstract

**Introduction:**

One of the main obstacles in providing effective treatments for major depressive disorder (MDD) is clinical heterogeneity, whose neurobiological correlates are not clearly defined. A biologically meaningful stratification of depressed patients is needed to promote tailored diagnostic procedures.

**Objectives:**

Using structural data, we performed an unsupervised clustering to define clinically meaningful clusters of depressed patients.

**Methods:**

T1-weighted and diffusion tensor images were obtained from 102 MDD patients. In 64 patients, clinical symptoms, number of stressful life events, severity and exposure to adverse childhood experiences were evaluated using the Beck Depression Inventory (BDI), Schedule of Recent Experiences (SRE), Risky Family Questionnaire (RFQ), and Childhood Trauma Questionnaire (CTQ). Clustering analyses were performed with extracted tract-based fractional anisotropy (TBSS, FSL), cortical thickness, surface area, and regional measures of grey matter volumes (CAT12). Gaussian mixture model was implemented for clustering, considering Support Vector Machine (SVM) as classifier. A 10x2 repeated cross-validation with grid search was performed for hyperparameters tuning and clusters’ stability. The optimal number of clusters was determined by normalized stability, Akaike and Bayesian information criterion. Analyses were adjusted for total intracranial volume, age, and sex. The clinical relevance of the identified clusters was assessed through MANOVA, considering domains of clinical scales as dependent variables and clusters’ labels as fixed factors. Discriminant analysis was subsequently performed to assess the discriminative power of these variables.

**Results:**

Cross-validated clustering approach identified 2 highly stable clusters (normalized stability=0.316, AIC=-80292.48, BIC=351329.16). MANOVA showed a significant between-clusters difference in clinical scales scores (p=0.038). Discriminant analysis distinguished the two clusters with an accuracy of 78.1%, with BDI behavioural and CTQ minimisation/denial domains showing the highest discriminant values (0.325 and 0.313).

**Conclusions:**

Our results defined two biologically informed clusters of MDD patients associated with childhood trauma and specific clinical profiles, which may assist in targeting effective interventions and treatments.

**Disclosure of Interest:**

None Declared

